# Adjunctive Pessary Therapy after Emergency Cervical Cerclage for Cervical Insufficiency with Protruding Fetal Membranes in the Second Trimester of Pregnancy: A Novel Modification of Treatment

**DOI:** 10.1155/2015/185371

**Published:** 2015-08-27

**Authors:** Katarzyna Kosinska-Kaczynska, Dorota Bomba-Opon, Aleksandra Zygula, Bartosz Kaczynski, Piotr Wegrzyn, Miroslaw Wielgos

**Affiliations:** ^1^1st Department of Obstetrics and Gynecology, Medical University of Warsaw, Plac Starynkiewicza 1/3, 02-015 Warsaw, Poland; ^2^Department of Medical Informatics and Telemedicine, Medical University of Warsaw, Ulica Banacha 1A, 02-097 Warsaw, Poland

## Abstract

*Aim*. To evaluate the effectiveness of adjunctive pessary therapy after emergency cervical cerclage (ECC) in improving perinatal outcome in cervical insufficiency with fetal membranes protruding into the vagina.* Material and Methods*. A retrospective analysis of patients treated at the 1st Department of Obstetrics and Gynecology, Medical University of Warsaw, between 2008 and 2013. The study group consisted of 15 women treated with ECC and a pessary and the control group consisted of 17 patients treated with cerclage only.* Results*. The mean gestational age at delivery was significantly higher in the study group (34.7 versus 29.7 weeks, *p* = 0.03). The period between cerclage insertion and delivery was significantly longer in the study group (82.9 versus 52.1 days, *p* = 0.045). The mean neonatal birthweight and neonatal “discharge alive” ratio were higher in the study group, although not statistically significant (2550 g versus 1883 g, *p* = 0.14, and 93.3% versus 70.5%, *p* = 0.18, resp.). NICU hospitalization rates were comparable (33.3% versus 35.3%, *p* = 0.9).* Conclusions*. Adjunctive pessary therapy allows delaying delivery in women treated with ECC due to cervical insufficiency with protruding fetal membranes. It also seems to improve neonatal outcome, although the differences are not statistically significant. Further prospective study is required to prove these findings.

## 1. Introduction

Cervical insufficiency is defined as asymptomatic cervical shortening and dilatation with the absence of detectable uterine contractions [1]. It affects about 0.05–1% of all pregnant women and is responsible for 15–25% of perinatal losses in the second trimester [2, 3]. In sporadic cases cervical insufficiency is unexpectedly diagnosed during the second trimester, when the cervix is found dilated, with or without fetal membranes protruding into the vagina. In such cases emergency (rescue) cervical cerclage (ECC) is usually performed.

Cervical cerclage is a treatment consisting of a strong suture placed around the cervix. It was first proposed by Shirodkar in 1955 [4]. A single encircling suture was placed at the internal os level after the dissection of the bladder. A later suture technique modification by McDonald simplified the procedure as the bladder dissection was no longer necessary. Another popular cerclage technique is Wurm suture. It consists of two perpendicular stitches placed around the cervix. Depending on the severity of the cervical insufficiency and the duration of pregnancy, cerclage insertion is called elective or urgent. When the cervix is found dilated during the second trimester, an emergency cerclage is often inserted. Although data concerning the efficacy of ECC are limited, it is suggested that ECC does delay delivery and improves the outcome [5–7]. According to retrospective cohort studies, ECC allows the prolongation of pregnancy and a longer gestational age at the delivery compared with bed rest [5–8]. Women treated with ECC required shorter hospitalization and less tocolysis and experienced fewer episodes of preterm rupture of membranes with no difference in the occurrence of chorioamnionitis [5]. ECC results in higher neonatal birthweight [5, 6, 8]. A prospective study on cervical incompetency with protruding membranes was published in 2006 by Daskalakis et al. [9]. It also revealed a significant prolongation of pregnancy and a higher neonatal birthweight in ECC group. ECC treatment was related to a higher rate of live births, higher neonatal survival, lower rate of preterm delivery before 32 weeks, and fewer Neonatal Intensive Care Unit admissions. All the above-mentioned studies proved a higher effectiveness of ECC over bed rest. As there is no other effective treatment of advanced cervical insufficiency, ECC may be the only method to delay delivery until fetal viability is reached [8].

Vaginal pessary is an effective tool in urinary incontinence and pelvic organ prolapse treatment. Arabin cervical pessary is used in preventing preterm delivery. It is a flexible, ring-shaped pessary designed to encompass the insufficient cervix. This option is less invasive than a cervical suture and its effectiveness in preventing preterm labour was established in several studies. Goya et al. conducted a randomized multicenter trial involving 385 pregnant women. The study showed that a cervical pessary significantly decreased the rate of preterm deliveries and neonatal complications [10]. Similar results were also presented in the Cochrane review [11]. Nowadays, a pessary is often used in cervical insufficiency treatment but it has never been used as an adjunctive tool after ECC insertion.

The aim of this study was to evaluate the effectiveness of adjunctive pessary therapy after emergency cervical cerclage insertion in improving perinatal outcome in women with cervical insufficiency with fetal membranes protruding into the vagina.

## 2. Material and Methods

We retrospectively analyzed the medical records of singleton pregnancy patients with cervical insufficiency treated with ECC due to cervical dilatation of up to 4 cm accompanied by bulging of fetal membranes into the vagina, diagnosed in the second trimester of pregnancy, hospitalized at the 1st Department of Obstetrics and Gynecology, Medical University of Warsaw, between 2008 and 2013. The inclusion criteria included intact membranes, no signs of intrauterine infection (maternal fever, uterine tenderness, foul-smelling vaginal discharge, fetal tachycardia and maternal leukocytosis, or elevated C-reactive protein), no uterine contractions, and no vaginal bleeding. They were fulfilled by 40 patients.

Cervical insufficiency was diagnosed during an examination with a vaginal speculum. High vaginal and/or cervical swabs were taken for microbiological culture. During an observation period lasting 12–24 hours the patients' general condition was checked, routine fetal ultrasound scan was performed, and white blood cell count (WBC) and C-reactive protein (CRP) levels were measured to diagnose subclinical chorioamnionitis. If the above results were normal (no regular uterine contractions, no fetal abnormalities detected on ultrasound scan, WBC ≤ 15 000 × 10^6^/L, and CRP < 10 mg/L) the ECC procedure was performed. No amniocentesis to exclude infection was performed before the ECC. In every case the patient's written informed consent was obtained.

A prophylactic dose of antibiotics was administered (3.0 g ampicillin and sulbactam and 0.5 g metronidazole intravenously) 30 minutes before the procedure. After the anesthesiologist administered spinal or general anesthesia, the patient was placed in the lithotomy position, and the vulva and the vagina were carefully decontaminated (Octenisept, Schulke & Mayr). The urinary bladder was filled with 0.9% NaCl through a Foley catheter in order to place protruding fetal membranes in the uterine cavity. The method of cerclage insertion (Wurm or McDonald technique, Ethibond Excel Number 2 Polyester Suture, Johnson) was selected individually at the discretion of the operator. On the day of the procedure 0.5 g of metronidazole was administered intravenously every 8 hours, and for the next 7 days 0.5 g of erythromycin was given orally every 8 hours, unless alternative therapy was chosen based on antibiogram. To avoid possible uterine contractions, intravenous tocolysis with fenoterol was administered for maximum 12 hours after the procedure (0.024–0.048 mg/min). Vaginal progesterone was administered (200 mg daily in two doses) to all patients beginning on the day after the procedure. Within 72 hours 8 patients revealed signs of infection, uterine contraction, or rupture of membranes. 15 out of the remaining 32 women were subsequently treated with a pessary (Herbich cervical pessary size 2). Adjunctive pessary treatment was left at the discretion of the attending obstetrician. Hence, the study group consisted of 15 women treated with cervical cerclage and an adjunctive pessary and the control group of 17 patients treated with cerclage only.

After at least 7 days of hospitalization, the patients were discharged home with no bed rest prescribed. Vaginal progesterone was administered until 34 weeks of gestation. In case of uterine contractions or vaginal bleeding the cerclage (and the pessary in the study group) was removed. In case of premature rupture of membranes the cerclage (and the pessary, if applicable) was removed if any signs of intrauterine infection or spontaneous uterine contractions were present. In case of such complications women were also administered steroids before the delivery (4 doses of 6 mg dexamethasone during 48 hours between 24 and 34 weeks of gestation). If no complications appeared, the cerclage (and the pessary, if applicable) was removed routinely at week 36 of pregnancy.

Patients' demographic and baseline test results were compared between the groups (age, parity, obstetric history, cervical dilatation, WBC, and CRP). The primary outcomes were as follows: the gestational week at late miscarriage or delivery and the prolongation of pregnancy after ECC procedure in both groups. The secondary outcomes were as follows: the mode of delivery and neonatal outcomes (neonatal survival, birthweight, and general condition according to the Apgar score and Neonatal Intensive Care Unit hospitalization).

Statistical analysis was performed with the Mann-Whitney *U* test for continuous variables and Fisher's test for categorical variables. A Kaplan-Meier curve was used to visualize time courses. Potential associations with the prolongation of pregnancy ≥28 weeks and ≥34 weeks were explored using a logistic regression analysis and reported as OR and 95% CI. Statistica 10.0 was used for statistical analyses. *p* values of <0.05 were considered significant and all tests were two-tailed.

## 3. Results

Basic characteristics of the groups are presented in Table 1. There were no statistical differences in age, parity, obstetric history, cervical dilatation, or WBC and CRP levels between the groups. After the removal of the cerclage all patients delivered before possible time of pessary insertion and thus were excluded from the study. The study group consisted of 15 women treated with cervical cerclage and an adjunctive pessary and the control group of 17 patients treated with cerclage only.

Before the cerclage 8 patients in the study group and 9 patients in the control group had high vaginal or cervical swabs taken for the microbiological culture. In the study group the results revealed 3 cases of* Enterococcus faecalis* invasion and single cases of* Escherichia coli*,* Streptococcus agalactiae*,* Staphylococcus aureus*,* Klebsiella pneumoniae*, and* Candida albicans*. Three cases of* Escherichia coli* and* Enterococcus faecalis* and one case of* Candida albicans* were reported in the control group. Other cultures revealed lactobacilli spp.

The average time of cerclage insertion in the study group was 22.7 weeks of gestation (from 20 to 25), which did not differ significantly from the control group (mean 22.1; min 19–max 27, *p* = 0.24). Cerclage according to Wurm technique was performed in 8 study group patients and McDonald technique in 7 study group patients. In the control group 10 women were treated with Wurm cerclage and 7 with McDonald cerclage (*p* = 0.7). After the procedure intravenous tocolysis was administered to 8 patients in the study group (53.3%) and 10 in the control group (58.8%, *p* = 0.77). Two patients in the study and three in the control group underwent antibiotic therapy according to the antibiogram. Antibiotics were administered after routine prophylactic course of ampicillin with sulbactam and metronidazole (cephalexin, clindamycin, or ciprofloxacin instead of erythromycin). Steroids were administered to 8 women in the study and 10 in the control group (53.3% versus 58.8%, *p* = 0.77).

**Figure 1:**
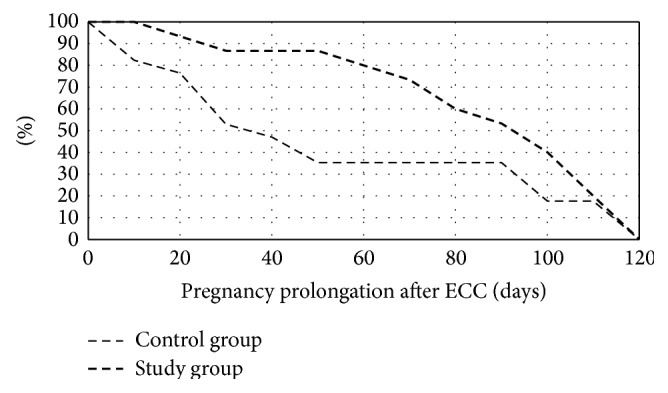
The percentage of women who did not deliver in the following days after the ECC procedure in both groups.

The comparison of pregnancy outcomes between the study group and the control group is presented in Table 2. None of the women had serious procedure-related complications. The mean gestational age at delivery was significantly higher in the study group (34.7 weeks versus 29.7 weeks in the control group, *p* = 0.03). The period between cerclage insertion and delivery was also significantly longer in the study group (82.9 versus 52.1 days, *p* = 0.045). The percentage of women who did not deliver in the following days after the ECC procedure in both groups is illustrated in Figure 1.

The mean neonatal birthweight was higher in the study group, but the difference was not significant (2550 g versus 1883 g, *p* = 0.14). In the study group 11 neonates were born in good (Apgar score 8–10 points), 2 were born in average (4–7 points), and 1 was born in poor general condition (0–3 points) and there was one case of stillbirth (at 22 weeks). In the control group 11 neonates were born in good, 1 was born in average, and 4 were born in poor general condition, and there was also one case of stillbirth (at 24 weeks). Two neonates born in poor general condition before 28 weeks of gestation died during the first 48 hours after the delivery. NICU hospitalization rates were comparable (33.3% versus 35.3%, *p* = 0.9). Neonatal “discharge alive” ratio was insignificantly higher in the study group (93.3% versus 70.5%, *p* = 0.2).

In a linear regression model primiparity, late miscarriage or preterm delivery in previous pregnancy, cervical dilatation ≥2 cm, and adjunctive pessary therapy were not significantly associated with the prolongation of pregnancy above 28 or 34 weeks (Table 3). Only the adjunctive pessary therapy was associated with the prolongation of pregnancy above 28 weeks with OR 5.9 and with *p* close to significance (*p* = 0.06).

## 4. Discussion

Cervical insufficiency remains a very serious pregnancy complication. In case of a history of second-trimester pregnancy loss or preterm delivery intensive screening and prophylactic procedures may be applied. But according to our results, as well as those presented in the literature, more than half of the patients with advanced cervical insufficiency unexpectedly diagnosed during the second trimester of pregnancy were primiparous [12]. Severe cervical incompetence is commonly found in women, mostly primiparous, with no ascertainable risk factors. In such cases ECC is usually performed.

The majority of data concerning ECC effectiveness come from retrospective analyses [6–8, 12–16], and only a few studies were conducted prospectively [5, 9, 17]. Although data from the literature are scarce, the insertion of ECC was proved to improve pregnancy outcome in comparison with bed rest [5–7]. In our study we tested a hypothesis that adding a pessary as an adjunctive tool after ECC placement may improve the outcome.

The primary clinical conditions of ECC insertion in our study were comparable to those previously published by other authors. The ECC was inserted on average at 22 weeks of gestation, similarly to procedures discussed in the literature, mostly between 21 and 23 weeks [7, 8, 12, 14–16]. Fetal membranes protruding into the vagina and intrauterine infection are the strongest predictors of unfavorable perinatal outcome [18]. In order to create two comparable groups of patients, we included only pregnant women with fetal membranes bulging into the vagina, with no signs of infection, who did not deliver during the first 72 hours after the ECC procedure. We compared pregnancy outcomes in both analyzed groups with previously published data. According to the literature the mean gestational age at delivery after ECC insertion ranged from 25 to 34 weeks [5, 7, 8, 13, 14, 17, 19]. Most authors reported the average prolongation of pregnancy between 5 and 13 weeks [6–9, 12–14, 16, 20–23], although much shorter periods were also reported [24, 25]. The above-mentioned differences were probably due to the diversity of studied groups of patients and a different methodology. In our study the mean prolongation of pregnancy was approximately 7.5 weeks in the control group, while in the study group it was significantly longer (over 11.5 weeks). Different authors reported a 50–67% rate of deliveries after 28 weeks, a 44–69% rate of deliveries after 32 weeks, and a 34–44% rate of deliveries after 36 weeks of gestation [7, 8, 12, 15, 19, 23]. We observed similar rates in the control group (52.9% of deliveries took place after 28 weeks, 47% after 32 weeks, and 35.3% after 34 weeks). In the study group the observed percentages were higher (93.3% of women delivered after 26 weeks, 86.7% after 28 weeks, and 66.7% after 34 weeks) and the differences in delivery rates after 26 and 32 weeks of gestation were statistically significant. As the pregnancy outcome was better in the study group than in the control group, which is consistent with outcomes previously reported in the literature, our novel approach seems to be beneficial for patients with ECC.

We observed an analogous relationship in the neonatal outcome. Total neonatal survival rate reported in the literature varied between 50% and 74% [6, 12, 14, 23, 25], and it was similar in our control group, while in the study group the survival rate was much higher. The rate of NICU admission was similar to the one reported in the literature [8, 9, 12, 14].

Possible adjunctive therapies after ECC discussed in the literature included antibiotics and progestogens, but no pessary. According to Romero et al., intrauterine microbiological invasion occurred in about 50% of women with asymptomatic cervical dilatation [26, 27]. The usefulness of amniocentesis to exclude intrauterine infection prior to ECC or antibiotic therapy in cerclage recipients is still discussed in the literature [28]. In our study no amniocenteses were performed, and all the patients were treated with antibiotics. There are insufficient data to support or discourage prolonged antibiotic use in ECC patients. However, there is some evidence that antibiotics administered after the cerclage may improve perinatal outcome [20]. In many studies antibiotic therapy was also applied [7–9, 12, 13, 16].

Progesterone administration appears to reduce uterine contractions due to its anti-inflammatory mechanisms, oxytocin inhibition, or improvement of immune function. Adjunctive progesterone therapy has not yet proved to be effective in ECC patients, but there are some data suggesting that it may be beneficial [29]. Progesterone or 17-OH progesterone therapy was also conducted in other studies [12, 16].

Our study is limited by its retrospective nature, lack of randomization, a small sample size, and selected study group, but this is the first report that evaluates the impact of adjunctive pessary therapy on perinatal outcomes in women with ECC. Although the study has its limitations, the presented preliminary results are promising. Vaginal pessary as an addition to the cerclage may be another step in improving perinatal outcomes in women with cervical insufficiency and protruding fetal membranes. However, further prospective randomized study is required to prove it.

## 5. Conclusion

Adjunctive pessary therapy allows delaying delivery in women treated with ECC due to cervical insufficiency with protruding fetal membranes. It also seems to improve neonatal outcome, although the differences are not statistically significant. Further prospective study is required to prove these findings.

## Figures and Tables

**Table 1:** Characteristics of the study and control group.

	Study group			Control group			
	*n* = 15			*n* = 17			
	Median (min–max)	Mean	SD	Median (min–max)	Mean	SD	*p*
Age	32 (22–40)	31.8	5.3	31 (21–38)	31.7	3.9	0.9

Primiparity^*∗*^	8 (53.3)			11 (64.7)			0.7

Prior preterm delivery^*∗*^	1 (6.7)			3 (17.7)			0.6

Prior second-trimester pregnancy loss^*∗*^	2 (13.3)			2 (11.8)			0.9

Pregnancy week at ECC	23 (20–25)	22.7	1.7	21 (19–27)	22.1	2.1	0.24

WBC (10^9^/L)	10.5 (9.3–14.9)	10.9	1.9	10.8 (8–14.8)	11.2	2.4	0.7

CRP (mg/L)	3.5 (1.2–9.3)	4.9	3.6	3 (0.8–8.7)	3.3	2.7	0.2

Cervical dilatation ≤2 cm^*∗*^	11 (73.3)			13 (765)			0.9

^*∗*^ *n* (%).

ECC: emergency cervical cerclage insertion.

WBC: white blood cell count.

CRP: C-reactive protein.

**Table 2:** Comparison of the pregnancy outcomes between the study and the control group.

	Study group			Control group			
	*n* = 15			*n* = 17			
	Median (min–max)	Mean	SD	Median (min–max)	Mean	SD	*p*
Gestational age at delivery (weeks)	35 (22–41)	34.7	6.3	28 (21–38)	29.7	6.8	0.03

Prolongation of pregnancy (days)	91 (12–119)	82.9	39	35 (5–119)	52.1	43.7	0.045

Miscarriage or preterm delivery^*∗*^	8 (53.3)			11 (64.7)			0.7

Miscarriage^*∗*^	0			2 (11.8)			0.5

Delivery >26 wks^*∗*^	14 (93.3)			10 (58.8)			0.041

Delivery >28 wks^*∗*^	13 (86.7)			9 (52.9)			0.06

Delivery >32 wks^*∗*^	13 (86.7)			8 (47.1)			0.028

Delivery >34 wks^*∗*^	10 (66.7)			11 (35.3)			0.9

PROM^*∗*^	6 (40)			5 (29.4)			0.7

Intrauterine infection^*∗*^	3 (20)			5 (29.4)			0.69

Spontaneous regular uterine contractions^*∗*^	7 (46.7)			8 (47.1)			0.9

Vaginal delivery^*∗*^	12 (80)			13/15 (86.7)			0.95

Cesarean delivery ^*∗*^	3 (20)			2/15 (13.3)			0.9
Indications							
Fetal malpresentation	2			2			
Imminent fetal asphyxia	1			0			

Neonatal birthweight (g)	2850 (540–3700)	2550	1021	1650 (520–3680)	1883	1194	0.14

Stillbirths^*∗*^	1 (6.7)			1 (5.9)			0.98

NICU admission^*∗*^	5 (33.3)			6 (35.3)			0.9

“Discharged alive”^*∗*^ rate	14 (93.3)			12 (70.5)			0.2

^*∗*^ *n* (%).

wks: weeks of gestation.

PROM: premature rupture of membranes.

NICU: Neonatal Intensive Care Unit.

**Table 3:** Odds ratios for potential associations for perinatal outcome in women after ECC.

	Delivery ≥28 weeks of gestation OR; 95% CI; *p*	Delivery ≥34 weeks of gestation OR; 95% CI; *p*

Primiparity	1.35; 0.22–8.37; 0.73	0.54; 0.1–2.76; 0.44

Late miscarriage or preterm delivery in previous pregnancy	2.55; 0.28–22.92; 0.38	2.56; 0.36–18.21; 0.33

Cervical dilatation ≥2 cm	1.2; 0.15–8.28; 0.86	0.71; 0.11–4.52; 0.7

Adjunctive pessary therapy	5.93; 0.84–41.95; 0.062	3.6; 0.69–18.95; 0.11
